# Gender-Specific Differences in Depressive Behavior Among Forensic Psychiatric Patients

**DOI:** 10.3389/fpsyg.2021.639191

**Published:** 2021-08-19

**Authors:** Judith Streb, Elena Ruppel, Anne-Maria Möller-Leimkühler, Michael Büsselmann, Irina Franke, Manuela Dudeck

**Affiliations:** ^1^Department of Forensic Psychiatry and Psychotherapy, Ulm University, Ulm, Germany; ^2^Department of Psychiatry and Psychotherapy, Ludwig-Maximilians-University, Munich, Germany; ^3^Department of Forensic Psychiatry, Psychiatric Services of Grisons, Chur, Switzerland

**Keywords:** depressive symptoms, gender differences, suicide, forensic psychiatric patients, suicide attempt

## Abstract

**Background:**

Women are almost twice as likely to develop depression than men, but men commit suicide more often. One explanation for this paradox is that current depression inventories do not fully capture typical male symptoms of depression. Several studies showed that most depression symptoms in men are masked by externalizing behaviors, such as aggressiveness, addiction, and risky behavior. Here, we explored the differences in depression symptoms between men and women in a forensic psychiatric sample.

**Methods:**

We screened 182 forensic psychiatric patients and selected a matched sample (21 women and 21 men). External symptoms of depression were assessed with the Gender-Sensitive Depression Screening (GSDS) and internal symptoms with the Beck Depression Inventory Revision.

**Results:**

Although externalizing behaviors were similar in both groups, we found a significant relationship between external and internal depression symptoms only in men. In addition, male forensic patients with a history of suicide had higher scores in the GSDS, whereas female patients with a history of suicide had higher scores in the Beck Depression Inventory Revision.

**Discussion:**

The finding that the GSDS detected depression symptoms in men indicates that this instrument might be useful for developing assessments to prevent suicide in forensic practice.

## Introduction

Depressive disorders are among the most common mental disorders in Western society (Müters et al., 2013). Epidemiological studies show that the lifetime prevalence of depression is two to three times higher in women than in men (Angst et al., 2002; Busch et al., 2013; Boysen et al., 2014; Jacobi et al., 2014, 2016; Hasin et al., 2018). Paradoxically, the risk of suicide is consistently three times higher in men than in women [World Health Organization (WHO), 2019]. If one assumes that not all, but a large number of suicides are a direct consequence of a depressive disorder, one must conclude that depression is underdiagnosed and undertreated in men (Wålinder and Rutz, 2001; Möller-Leimkühler et al., 2007; Möller-Leimkühler, 2010).

Studies have identified many reasons why rates of diagnosis and treatment are lower in men (Warren, 1983; Fava et al., 1991; Spence and Robbins, 1992; Courtenay, 2000; Möller-Leimkühler, 2000, 2002, 2016; Swendsen and Merikangas, 2000; Möller-Leimkühler et al., 2002; Zierau et al., 2002; Cochran and Rabinowitz, 2003; Yu et al., 2004; Berger et al., 2005; Brownhill et al., 2005; Kessler et al., 2005; Sigmon et al., 2005; Winkler et al., 2005; Emslie et al., 2006; Rutz and Rhimer, 2007; Davis et al., 2008; Hausmann et al., 2008; Magovcevic and Addis, 2008; Oliffe and Phillips, 2008; Chuick et al., 2009; Cohn et al., 2009, 2010; Levant et al., 2009, 2011, 2013; Rochlen et al., 2010; Weaver et al., 2010; Martin et al., 2011, 2013; McCusker and Galupo, 2011; Oliffe et al., 2011, 2012, 2013; Rice et al., 2013; Lai et al., 2015; Yousaf et al., 2015; Whittle et al., 2015; Seidler et al., 2016; Cavanagh et al., 2017; Reiß, 2017; Rommel et al., 2017; Braly et al., 2018; Keil et al., 2020; Serafini et al., 2016, 2017). For example, men use medical services and preventive and health-promoting measures far less often than women (Seidler et al., 2016; Rommel et al., 2017). This behavior is even more pronounced in men with mental disorders (Keil et al., 2020). In this context, research found that men’s attitude toward seeking professional help depends on their conformity with masculine norms (Berger et al., 2005; Levant et al., 2009, 2011, 2013; McCusker and Galupo, 2011; Yousaf et al., 2015). The masculine role model implies competence, performance, and success. However, mental disorders, especially depression, are often accompanied by feelings of powerlessness, helplessness, and loss of control. These feelings are in contrast to masculine role expectations and can lead men to perceive depression as a failure in their role as a man (Warren, 1983; Courtenay, 2000; Emslie et al., 2006). To counteract this perception and protect their masculine identity against social discrimination, men often deny and distance themselves from depression or try to hide it from others (Sigmon et al., 2005; Hausmann et al., 2008; Möller-Leimkühler, 2016). As a consequence, they do not show prototypical symptoms of depression (e.g., sadness, crying, and hopelessness) to the outside world (Möller-Leimkühler, 2000, 2002).

Instead of seeking help, men often rely on self-medication with non-prescription substances, and their use of alcohol and drugs is particularly widespread (Möller-Leimkühler et al., 2002; Chuick et al., 2009; Rochlen et al., 2010; Oliffe et al., 2012, 2013; Reiß, 2017). This increased alcohol and substance use is reflected on the one hand by the high comorbidity between the disorders of depression and alcohol dependence (Swendsen and Merikangas, 2000; Kessler et al., 2005; Davis et al., 2008; Lai et al., 2015) and on the other hand by the finding that men with depression consume far more alcohol than women with depression (Angst et al., 2002; Martin et al., 2013; Cavanagh et al., 2017). Alcohol consumption often represents an escape from stressful situations, with the aim of suppressing and numbing the negative emotions (Chuick et al., 2009; Rochlen et al., 2010; Oliffe et al., 2011, 2012, 2013). Not only do men use alcohol and drugs to escape and avoid depression, but they also resort to other addictive behaviors. For example, men with depression show an increased focus and overcommitment in their professional lives, referred to as being a workaholic, particularly frequently (Spence and Robbins, 1992; Möller-Leimkühler, 2002; Cochran and Rabinowitz, 2003; Rutz and Rhimer, 2007; Oliffe and Phillips, 2008; Oliffe et al., 2013; Whittle et al., 2015). In numerous studies, men also report aggressiveness and outbursts of anger during a depressive episode (Fava et al., 1991; Zierau et al., 2002; Winkler et al., 2005; Magovcevic and Addis, 2008; Martin et al., 2013; Rice et al., 2013). In men, aggressive behavior is an accepted way to resolve conflicts and is seen as a practical way to regain control over negative feelings (Cohn et al., 2009, 2010; Weaver et al., 2010; Braly et al., 2018). Moreover, aggressiveness is firmly anchored in the masculine role model. Depressive men also show increased risky behavior (Brownhill et al., 2005; Martin et al., 2013; Cavanagh et al., 2017). For example, a study by Yu et al. (2004) observed dangerous driving behavior (fast driving, rapid acceleration, frequent lane changing, running red lights, and driving when tired) in men with depression or alcohol misuse.

The coping strategies listed above usually only help in the short-term, and over a longer period of time rather lead to an aggravation of the depressive symptoms and an intensification of stress. For many men, suicide is therefore the last option to cope with depression (Brownhill et al., 2005; Möller-Leimkühler, 2009; Armstrong et al., 2020). Suicide “appears to be the last resort to save self-worth and maintain the illusion of self-determination and autonomy of action − this only succeeds if the suicide attempt is violent and ends fatally” (Möller-Leimkühler, 2009) (authors’ translation).

To sum up, studies show that in men the prototypical symptoms of depression are often masked by externalizing behaviors, such as alcohol consumption, aggressiveness, and risky behavior. However, these symptoms are not included in either the standard depression inventories or the diagnostic criteria for depression in the Diagnostic and Statistical Manual of Mental Disorders (DSM) or the International Classification of Diseases (ICD), making it difficult to diagnose depression in men. The symptoms listed in conventional depression inventories like the BDI-R cover somatic (e.g., “I have no appetite at all”), affective (e.g., “I cry at the slightest occasion”), and cognitive (e.g., “I can’t concentrate anymore”) domains. In particular, the affective domain tends to be sensitive to women, since they are predominantly based on symptom descriptions by women that reflect internalizing coping forms of depressive experiences, such as brooding, crying, depressive moods, apathy, or loss of drive and interest. However, these affective symptoms, which are considered prototypical, are consistently less frequently reported by men with depressive disorders, so that they often do not meet the threshold for clinical depression and thus fall off the diagnostic grid (Martin et al., 2011). To solve this problem, over the past two decades researchers in English-speaking countries have developed various gender-sensitive depression scales that are intended to capture these externalizing behaviors in depressed men (Zülke et al., 2018; Rice et al., 2020). The first German-language depression scale that includes both classic depression symptoms and patterns of externalizing behavior specific to men, the Gender-Sensitive Depression Screening (GSDS-25), was recently developed by Möller-Leimkühler and Mühleck (2020). Externalizing symptoms, such as increased irritability, aggressiveness, outbursts of anger, hyperactivity, or addictive and risky behaviors, are not symptoms of a depressive disorder, since they serve men as a coping strategy to maintain the male role model. However, they can be used as indicators, in the sense of a clue, to diagnose depressive disorders in men. GSDSs capture a broader range of behaviors and should better identify depression risk in men.

In Germany, admission to a forensic psychiatric hospital follows a court decision according to Section 64 of the German criminal code. If a person has committed a serious offense as a result of a substance use disorder and has a high risk of reoffending and a favorable treatment prognosis, the court orders that the person be placed in a forensic psychiatric hospital. Therefore, we decided to study a forensic sample to examine whether externalizing behavior is associated with depressive symptoms in men or whether this association can also be observed in women. In other words, we wanted to investigate whether men and women with substance use show aggressive and risky behavior and whether they try to suppress the negative feelings associated with depression. Forensic psychiatric patients admitted for treatment according to Section 64 of the German criminal code are particularly suitable for investigating this question because they have been diagnosed with substance misuse and show behaviors such as aggressive and risky behavior much more frequently than people in the general population. A second reason for conducting this study in a forensic psychiatric sample is that suicide rates are extremely high in this population. The suicide rate in the general population in Germany is 19.7 per 100,000 men and 7.7 per 100,000 women [World Health Organization (WHO), 2019], but it is significantly higher in closed institutions: in prison, the suicide risk is 96.9 per 100,000 male inmates and 60.8 per 100,000 female inmates (Meischner-Al-Mousawi et al., 2020), and in forensic psychiatric hospitals it is 163.0 per 100,000 male patients (separate data for women are missing; Voulgaris et al., 2018).

Despite the extremely high suicide risk among forensic psychiatric patients, no studies have included forensic psychiatric samples to better understand the clinical usefulness of screening for externalizing symptoms in addition to completing standard depression inventories in this population. The use of GSDSs in forensic psychiatric patients might contribute to the development of more effective prevention measures and subsequently decrease suicide attempts in forensic psychiatric hospitals. Therefore, the aim of the present study was to explore differences in depression symptoms between men and women in a forensic psychiatric sample of people with substance misuse. We hypothesized that externalizing behaviors are correlated with depressive symptoms in men but not in women. Since depressed women generally score higher on the BDI-R than depressed men, the samples under study were parallelized with respect to the BDI-R score so that each woman was assigned a man with a similarly high score. As a result, the men studied did not differ from the women in terms of BDI-R score, even though they generally score less highly. When men report depression symptoms, they come into conflict with the prevailing masculine role model. One coping strategy to protect masculine identity is to report particularly masculine behaviors. Therefore, we expect that men who report depressive symptoms in the BDI-R should also score higher in the GSDS. A similar strategy is not expected for the female group, as the social role model allows women to be weak, tearful, or sad. If the addition of externalizing behavior better detects depression in men because a broader spectrum is queried, the GSDS should be more strongly related to suicidal behavior than the BDI-R. This is not expected for the group of women.

## Materials and Methods

### Sample

A total of 182 forensic psychiatric patients aged from 19 to 79 years were asked to participate in the study [161 men and 21 women; mean (SD) age, 34.62 (11.24) years]. At the time of the survey, all participants were being treated in one of the 13 participating hospitals in the state of Bavaria, Germany. Inclusion criteria were age 18 years or older and ability to give informed consent in the opinion of the professionals responsible for their treatment. The exclusion criterion was the presence of acute symptoms of a psychotic disorder.

### Procedure

Patients were informed about the study objectives and that neither participation nor non-participation would have any advantages or disadvantages with respect to their treatment. Patients who agreed to participate gave written informed consent. Patients received neither financial nor non-financial compensation for their participation. They completed the questionnaires in small groups in a separate room on the ward, and a research assistant was available to provide help. The study was funded by the Bavarian State Ministry of Family, Job and Social Affairs, Germany; it was approved by the Ethics Committee of the University of Ulm, Germany (application number: 174/17) and was performed in accordance with the Declaration of Helsinki.

### Materials

In a first step, participants completed a questionnaire to collect sociodemographic (age, sex, and highest level of education), clinical (main diagnosis and prior suicide attempts), and legal data (index offense, i.e., the offense that led to the current admission). Then, they completed the GSDS (Zülke et al., 2018) and the Beck Depression Inventory Revision (BDI-II; Hautzinger et al., 2006).

The GSDS is a self-assessment tool that assesses depressive symptoms, especially those found more often in men (Möller-Leimkühler and Mühleck, 2020). The patients were asked about symptoms in the 6 months before admission to hospital. The questionnaire includes both typical and atypical, predominantly male (external) depressive symptoms. The 26 items of the screening are divided into six subscales: internal depressive symptoms (example: I had little interest or pleasure in my daily activities), stress perception (example: I felt under time pressure), emotional control (example: I kept my feelings to myself), aggressiveness (example: I had outbursts of anger that I could not control), alcohol misuse (example: I thought about alcohol more often), and risky behavior (example: I endangered myself with my driving style). Each item is rated on a 4-point Likert scale ranging from *never* or *rarely* (= 0) to *mostly* or *always* (= 3). To evaluate the scores, we calculated the mean values of the subscales and the entire scale. The GSDS has proven good reliability (Cronbach’s alpha of total scale, *r* = 0.88; Cronbach’s alpha of subscales, from *r* = 0.86 to *r* = 0.70) and satisfactory convergent validity with the short version of the General Depression Scale (ADS-K, Spearman’s Rho = 0.79; Hautzinger and Bailer, 1993).

The BDI-II (Hautzinger et al., 2006) measures the severity of depressive symptoms and is the most well-established inventory for depression. It measures the severity of depressive (somatic, affective, and cognitive) symptoms with the help of 21 items, each of which is answered by selecting 1 of 4 statements (example: 0 = I do not feel sad. 1 = I feel sad. 2 = I am sad all the time and I can’t snap out of it. 3 = I am so sad or unhappy that I can’t stand it). As described in the manual, we calculated a total score for all items (maximum: 63 points). The authors of the scale specify the following cut-off values: 0–8, no depression; 9–13, minimal depression; 14–19, mild depression; 10–18, moderate depression; and 29–63, severe depression [Beck et al., 1996; World Health Organization (WHO), 2017]. According to Hautzinger et al. (2006), the reliability is excellent, with values for Cronbach’s alpha between *r* = 0.89 and *r* = *0.93* across different samples. The correlation between BDI-II scores and other depression questionnaires shows satisfactory convergent validity (0.72 ≤ *r* ≤ 0.89 and 0.68 ≤ *r* ≤ 0.70).

### Statistical Analyses

First, we assigned all 21 female patients to 21 of the 161 male patients by a case-control matching procedure, controlling for the factors age (±4 years) and BDI-II score (±3 total points). The matched sample thus comprised 42 patients (21 male and 21 female). Statistical analyses were performed on the matched sample. Sociodemographic data and questionnaire scores were analyzed separately for men and women. To compare the groups, we used paired *t*-tests for metric variables and Chi-squared tests or Fisher’s exact tests for frequencies. Pearson correlations between the standard depression inventory (BDI-II) and the total mean value and the subscales of the GSDS were also computed separately for men and women. To compare the correlation coefficients in men and women, we tested them for significance according to the specifications of Eid et al. (2011)^1^. Finally, we compared the BDI-II and GSDS scores of patients with and without a history of suicide attempt by independent *t*-tests. Data were analyzed with IBM SPSS Statistics for Windows Version 25 (IBM Corp., Armonk, NY, United States).

## Results

We found no significant differences in sociodemographic variables between men and women (see Table 1).

**TABLE 1:** Sociodemographic data of an age- and BDI-II score-matched sample of male (*n* = 21) and female (*n* = 21) forensic psychiatric inpatients.

	Men	Women	Statistics
		
	*M* (SD)/*n* (%)	*M* (SD)/*n* (%)	
Age	35.95 (10.6)	36.43 (10.0)	*t*(20) = −0.872, *p* = 0.394
Highest level of education			FET = 5.413, *p* = 0.115
None	3 (14%)	0	
Secondary school	11 (52%)	9 (43%)	
Technical school	6 (29%)	7 (33%)	
High school	1 (5%)	5 (24%)	
Main diagnosis			FET = 2.766, *p* = 0.798
Substance use disorder	13 (62%)	14 (67%)	
Schizophrenia	4 (19%)	3 (14%)	
Personality disorder	4 (19%)	2 (10%)	
Affective disorder	0	1 (5%)	
Other disorders	0	1 (5%)	
Index offense^1^			FET = 3.051, *p* = 0.964
Robbery	2 (10%)	1 (5%)	
Assault	8 (40%)	6 (30%)	
Sexual crime	1 (5%)	1 (5%)	
Fraud	1 (5%)	1 (5%)	
Theft	2 (10%)	1 (5%)	
Arson	1 (5%)	1 (5%)	
Drug offense/narcotic substances	4 (20%)	7 (35%)	
Other crimes	1 (5%)	2 (10%)	
Prior suicide attempts	6 (28%)	11 (52%)	*X*^2^(1) = 2.471, *p* = 0.208

*BDI-II, Beck Depression Inventory Revision; *M*, mean; SD, standard deviation; FET, Fisher’s exact test. ^1^Missing data, men = 1, women = 1.*

Because the male and female samples were matched according to their BDI score, as expected we found no significant differences in the score between the two samples (see Table 2). In addition, the GSDS scores showed no significant differences between men and women in either the total mean score or the subscale scores, i.e., female forensic patients appeared to report exhibiting externalizing behaviors, such as aggressiveness and risky behavior, with the same frequency as men (Table 2).

**TABLE 2:** Descriptive statistics of the Beck Depression Inventory Revision (BDI-II) and Gender-Sensitive Depression Screening (GSDS) in an age- and BDI-II score-matched sample of male (*n* = 21) and female (*n* = 21) forensic psychiatric inpatients.

	Men	Women	Statistics
		
	*M* (SD) or *n* (%)	*M* (SD) or *n* (%)	
BDI-II depression severity (score)	13.71 (10.35)	15.00 (9.88)	*t*(20) = −1.477, *p* = 0.155, *d*_*Cohen*_ = 0.322
No depression (0–8)	8 (38%)	6 (29%)	FET = 0.913, *p* = 0.967, Cramer-*V* = 0.106
Minimal depression (9–13)	5 (24%)	6 (29%)	
Mild depression (14–19)	2 (10%)	2 (10%)	
Moderate depression (20–28)	5 (24%)	6 (29%)	
Severe depression (29–63)	1 (5%)	1 (5%)	
GSDS total mean value	1.06 (0.52)	1.26 (0.50)	*t*(20) = −1.448, *p* = 0.163, *d*_*Cohen*_ = 0.316
Internal depressive symptoms	0.93 (0.73)	1.33 (0.82)	*t*(20) = −1.889, *p* = 0.073, *d*_*Cohen*_ = 0.412
Aggressiveness	0.92 (0.89)	1.19 (1.12)	*t*(20) = −1.095, *p* = 0.286, *d*_*Cohen*_ = 0.239
Stress perception	1.08 (0.64)	1.36 (0.84)	*t*(20) = −1.033, *p* = 0.314, *d*_*Cohen*_ = 0.225
Emotional control	1.98 (0.70)	1.86 (0.94)	*t*(20) = 0.525, *p* = 0.605, *d*_*Cohen*_ = −0.115
Alcohol abuse	0.95 (1.00)	1.02 (1.06)	*t*(20) = −0.197, *p* = 0.846, *d*_*Cohen*_ = 0.043
Risky behavior	0.21 (0.68)	0.31 (0.73)	*t*(20) = −0.409, *p* = 0.687, *d*_*Cohen*_ = 0.089

*FET, Fisher’s exact test; *M*, mean; SD, standard deviation; BDI-II, Beck Depression Inventory Revision; GSDS, Gender-Sensitive Depression Screening.*

However, the correlation analysis between BDI-II and GSDS showed gender-specific differences. In the male patients, the mean BDI-II score showed significant positive correlations with the GSDS total mean value and the scores on the GSDS subscales depressive symptoms, aggressiveness, emotional control, and alcohol consumption (see Table 3). In the female patients, the BDI-II score did not correlate significantly with either the total mean GSDS score or the subscale scores (see Table 3). Although women reported externalizing behaviors, such as aggressiveness, alcohol misuse, and risky behavior, with the same frequency as men, these behaviors were not associated with typical depressive symptoms (measured by the BDI-II). Comparing the Fisher-*z* transformed correlation coefficients of men with those of women validated the observed differences.

**TABLE 3:** Pearson correlations between the Beck Depression Inventory Revision (BDI-II) and Gender-Sensitive Depression Screening (GSDS) total score and subscales in an age- and BDI-II score-matched sample of male (*n* = 21) and female (*n* = 21) forensic psychiatric inpatients.

	BDI-II score		
	Men	Women	Significance test for correlations
GSDS total mean value	0.691**	0.278	*z* = 1.693, *p* = 0.045
Depressive symptoms	0.731**	0.070	*z* = 2.582, *p* = 0.005
Aggressiveness	0.638**	0.243	*z* = 1.521, *p* = 0.064
Stress perception	0.143	0.367	*z* = −0.723, *p* = 0.235
Emotional control	0.469*	0.343	*z* = 0.454, *p* = 0.325
Alcohol consumption	0.492**	–0.289	*z* = 2.509, *p* = 0.006
Risk behavior	0.339	–0.076	*z* = 1.287, *p* = 0.099

Significance tests for correlations were performed according to Eid et al. (2011). BDI-II, Beck Depression Inventory Revision; GSDS, Gender-Sensitive Depression Screening. *p < 0.05, **p < 0.01.

Finally, we examined whether the BDI-II and GSDS depression scores were different between patients with and without a history of suicide. As can be seen in Table 4, men with a history of attempted suicide had higher total mean GSDS scores than men without such a history. The groups did not differ in the BDI-II. In the women, the result was the opposite, i.e., female patients with a history of suicide attempt had higher scores in the BDI-II than women without such a history, but a past suicide attempt had no influence on the GSDS score.

**TABLE 4:** Comparison of patients with and without a history of suicide attempt in an age- and BDI-II score-matched sample of male (*n* = 21) and female (*n* = 21) forensic psychiatric inpatients.

	Suicide attempt	No suicide attempt	
		
	*n* or *M* (SD)	*n* or *M* (SD)	Statistics
Men	6	15	
BDI-II score	15.80 (9.04)	14.27 (10.98)	*t*(19) = 0.346, *p* = 0.733, *d*_*Cohen*_ = 0.151
GSDS total mean value	1.52 (0.45)	1.04 (0.45)	*t*(19) = 2.440, *p* = 0.025, *d*_*Cohen*_ = 1.066
Women	11	10	
BDI-II score	20.83 (11.84)	10.87 (8.53)	*t*(19) = 2.169, *p* = 0.043, *d*_*Cohen*_ = 1.048
GSDS total mean value	1.18 (0.56)	1.02 (0.52)	*t*(19) = 0.642, *p* = 0.528, *d*_*Cohen*_ = 0.310

**M*, mean; SD, standard deviation; BDI-II, Beck Depression Inventory Revision; GSDS, Gender-Sensitive Depression Screening.*

## Discussion

The aim of this study was to explore internal and external symptoms of depression in a forensic psychiatric sample and to test the hypothesis that externalizing behaviors are correlated with depressive symptoms in men but not in women. The statistical analysis showed gender-specific differences between BDI-II and GSDS. In the male patients, the mean BDI-II score showed significant positive correlations with the GSDS score but in the female patients, the BDI-II score did not correlate significantly with the GSDS score. Our results confirmed previous investigations showing that dysfunctional coping strategies in depressed men are characterized by aggressiveness, alcohol use, and risky behavior (Möller-Leimkühler, 2005, 2009; Martin et al., 2013; Möller-Leimkühler and Mühleck, 2020). In contrast to male patients, female patients who exhibited external behaviors did not appear to have typical depressive symptoms because we found no significant correlations with the standard depression inventory BDI-II. Therefore, we suggest that in female forensic psychiatric patients aggressiveness, alcohol misuse, and risky behavior before admission to hospital might be related to other factors, such as certain criminogenic or social factors, but not to depression. The assumption that externalizing behavior *per se* is associated with depression could therefore be rejected. A significant correlation is only evident in men, so we could hypothesize that the effect is due to masculinity. The lack of an association between external behaviors and the scores on the standard depression inventory in women indicates that the GSDS might not be suitable for detecting depressive symptoms in female forensic psychiatric patients.

We suppose, that men could be ashamed to report affective symptoms to others because they do not want to appear weak and helpless. Instead, they could exhibit particularly masculine behaviors (drinking alcohol or being aggressive) that fit the male role norm. Thus, externalizing behaviors in men primarily serve masking or coping purposes. In the context of psychiatric diagnosis, externalizing behaviors could be used as additional indicators of a depressive disorder. However, externalizing behaviors are not *per se* indicators of a depressive disorder in men; they occur when men want to conform to the male role mode. In addition, the presence of related psychiatric disorders such as substance use disorder or dissocial personality disorder must be considered as a possible cause of the externalizing behaviors.

Several studies have established impulsivity, substance misuse, and aggressiveness as significant risk factors for suicidal behavior (Hillbrand, 1995; Mann et al., 1999; Stålenheim, 2001; Dudeck et al., 2016; Armstrong et al., 2020; Shafiee-Kandjani et al., 2020). Our data in male patients corroborate these findings because GSDS scores (but not standard depression inventories) were associated with previous suicidal behavior in men; thus, the early detection of external depression symptoms could contribute to establishing a precise prognosis and preventing suicidal attempts. Given the increased risk of suicidal behavior in forensic psychiatric patients, establishing preventive measurements is a high priority. The GSDS is a good instrument for identifying atypical external symptoms of depression in male forensic psychiatric patients and might be suitable for use as a standard evaluation instrument in forensic psychiatric settings. On admission to forensic psychiatry, the main focus of diagnostic assessment is to identify a substance use disorder, as this forms the basis of treatment. A depressive disorder as a secondary diagnosis is assigned extremely rarely (in the present sample, only one woman received this diagnosis). Nevertheless, 11 of 42 patients reported symptoms of moderate depression and two reported symptoms of major depression in the BDI-R. If the substance use disorder is merely a consequence of an underlying depressive disorder, the depression would have to be treated first and the addictive disorder only in a second step. In other words, doctors and therapists in forensic psychiatric hospitals should check for the presence of a depressive disorder – also in view of the high suicide risk of their patients – and take into account that men tend to mask their feelings. The present study illustrates the urgency of considering external behaviors when diagnosing depressive symptoms in men and of identifying further gender-specific risk factors for depression in forensic psychiatric populations. In addition to the use of suitable gender-sensitive depression scales, programs should also be developed to draw attention to male depression. For example, medical staff should be sensitized to these symptoms so that they can easily identify depression in men. Patients should also be informed about atypical symptoms of depression so that they can recognize these symptoms and seek professional support.

### Limitations

The results of the present study are based on 42 patient data and should be interpreted with caution. Replication of the study on a larger sample would be desirable. One further limitation of the present study is that we did not assess masculinity beliefs. In addition, self-reported data can result in various biases; for example, patients may give socially desirable responses. Although none of the participants received antidepressants during the study, the fact that we did not consider differences in medication (and side effects) can be seen as another limitation. Given that most of the forensic psychiatric patients in Germany are male, few female patients were available for this study, resulting in a small sample size. Another limitation is that there was no assessment of anti-social attitudes and other key criminogenic factors.

## Data Availability Statement

The raw data supporting the conclusions of this article will be made available by the authors, without undue reservation.

## Ethics Statement

The studies involving human participants were reviewed and approved by the Ethics Committee of the University of Ulm, Germany. The patients/participants provided their written informed consent to participate in this study.

## Author Contributions

MD, IF, and A-MM-L designed the study. MB collected the data. ER and JS analyzed and interpreted the data and wrote the initial draft of the manuscript. All authors had full access to all the data in the study and take responsibility for the integrity and accuracy of the data analysis. All authors contributed to, read and approved the final version of the manuscript.

## Conflict of Interest

The authors declare that the research was conducted in the absence of any commercial or financial relationships that could be construed as a potential conflict of interest.

## Publisher’s Note

All claims expressed in this article are solely those of the authors and do not necessarily represent those of their affiliated organizations, or those of the publisher, the editors and the reviewers. Any product that may be evaluated in this article, or claim that may be made by its manufacturer, is not guaranteed or endorsed by the publisher.

## Footnotes

1
https://www.psychometrica.de/korrelation.html
